# Power Doppler in hand joints predicts therapeutic failure in treatment-naive women with early rheumatoid arthritis: A prospective study

**DOI:** 10.1016/j.clinsp.2025.100593

**Published:** 2025-03-02

**Authors:** Karine Rodrigues da Luz, Jamil Natour, Marcelo de Medeiros Pinheiro, Giovanna S. Petterle, Marla Francisca dos Santos, Artur da Rocha Correa Fernandes, Rita Nely Vilar Furtado

**Affiliations:** a Disciplina de Reumatologia, Universidade Federal de São Paulo/Escola Paulista de Medicina (UNIFESP/EPM), São Paulo, SP, Brazil; b Departamento de Diagnóstico por Imagem, Universidade Federal de São Paulo/Escola Paulista de Medicina (UNIFESP/EPM), São Paulo, SP, Brazil

**Keywords:** Rheumatoid arthritis, Diagnostic imaging, Ultrasonography, Hand joints, Drug therapy

## Abstract

**Objective:**

This study aimed to determine whether ultrasound measurements of the hands could predict treatment failure in treatment-naive women with early rheumatoid arthritis.

**Method:**

In a prospective case-control study, 48 women underwent blind assessments four times over 48-weeks, considering three failure stages: failure 1 (methotrexate), failure 2 (leflunomide), and failure 3 (adalimumab). Bilateral ultrasound exams evaluated wrist, 2nd, and 3rd Metacarpophalangeal Joints (MCPs), and Proximal Interphalangeal Joints (PIPs) for inflammatory indicators (synovial and tenosynovial proliferation using grayscale and Power Doppler [PD]) and joint damage (bone erosion and cartilage damage).

**Results:**

The study involved 48 women, aged 47.7 ± 11.6 years, with an average disease duration of 7.5 ± 3.5 months. Of these, 41 (85.41 %) experienced failure 1, 25 (52 %) experienced failure 2, and 5 (10.5%) experienced failure 3. Predictors for failure 1 included PD/Q10 total score > 2.5 (OR = 18.00), PD/SQ10 total score > 5.0 (OR = 23.2), PD/Q MCP score > 1.5 (OR = 14.58), and PD/SQ MCP score > 3.0 (OR = 35). For failure 2, predictors encompassed PD/Q10 total score > 4.5 (OR = 4.81), PD/SQ10 total score > 9.5 (OR = 4.81), PD/Q MCP score > 2.5 (OR = 4.92), PD/SQ MCP score >5.0 (OR = 6.22), and PD/Q PIP score > 1.5 (OR = 6.66). In relation to failure 3, a PD/Q wrist score > 2.5 (AUC = 0.79; *p* = 0.035) was indicative.

**Conclusions:**

Power Doppler proved to be a predictive indicator for treatment failure in early rheumatoid arthritis among treatment-naive women. It emerged as a predictor for both the initial and 2nd DMARD treatments, as well as the 1st immunobiological treatment, based on hand joint assessments.

**Trial registration:**

Clinical trials.gov NCT04752748.

## Highlights

• Power Doppler as a Predictor: In hand ultrasound of women with early rheumatoid arthritis, Power Doppler was a significant predictor of therapeutic failures.
• Important Power Doppler Scores: The total Power Doppler score, wrist Power Doppler score, and metacarpophalangeal Power Doppler score were the most critical predictors of therapeutic failures.
• Clinical Implications of Power Doppler: The use of Power Doppler can help clinicians identify the risk of therapeutic failures in women with early rheumatoid arthritis, allowing for early intervention and treatment adjustments to improve patient outcomes.

## Introduction

Rheumatoid Arthritis (RA) is a potentially devastating inflammatory joint disorder. Currently, alterations in the therapeutic approach for RA patients typically rely on clinical and laboratory measurements, along with the computation of disease activity scores such as the 28-Joint Disease Activity Score (DAS28), Simplified Disease Activity Score (SDAI), and Clinical Disease Activity Index (CDAI).^1^, ^2^, ^3^

However, these clinical and laboratory scores may fall short as predictors of RA treatment response to disease-modifying anti-rheumatic drugs (DMARDs).^4^^,^^5^

The utilization of imaging techniques, such as joint Ultrasound (US), can aid in evaluating and monitoring these patients.^5^^,^^6^ There exists evidence demonstrating the impact of US employment in diagnosing early Rheumatoid Arthritis (ERA), characterized by an onset of <12-months. The US approach has demonstrated greater accuracy compared to clinical examinations in detecting subclinical synovitis, with Power Doppler (PD) facilitating the diagnosis of ERA patients experiencing joint pain alone, in addition to predicting structural damage.^6^, ^7^, ^8^ The US can also be valuable in overseeing RA treatment involving both DMARDs and immunobiological therapy.^9^, ^10^, ^11^, ^12^

Recently, a 10-joint score (US10) involving the hands and wrists has been shown to be both valid and reproducible for monitoring inflammation and joint damage in ERA patients, alongside displaying a significant correlation with clinical and laboratory findings.^13^ Despite demonstrating the potential for predicting future joint damage and relapse following clinical remission, only a limited number of studies have evaluated the capacity of the US to forecast therapeutic failure in RA or ERA.^14^, ^15^, ^16^

The objective of the current study was to assess whether joint Ultrasound (US) of the hands and wrists could predict therapeutic failure in a treatment regimen that included a first and second DMARD, as well as a first immunobiological drug, among ERA patients monitored over 48-weeks. Our primary Hypothesis (H1) was that there would be at least one baseline ultrasound measurement capable of predicting therapeutic failure after one year of treatment for these patients. Our null Hypothesis (H0) was that these ultrasound changes would not predict therapeutic failure during the follow-up period.

## Material and methods

### Patients

A case-control prospective study was undertaken, involving forty-eight consecutive patients with Early Rheumatoid Arthritis (ERA), characterized by a symptom duration of less than one year since onset. These patients were recruited from the Rheumatology Outpatient Clinics. This experimental protocol received approval from a local institutional review board (CEP 1061/08), and informed consent was obtained from all human subjects, in accordance with the World Medical Association Declaration of Helsinki: Ethical principles for medical research involving human subjects in 2013. The study was conducted between August 2014 and August 2016, adhering to the Helsinki Declaration, and was registered on clinicaltrials.gov (NCT04752748).

### Sample calculation

Considering the semi-quantitative score of total synovial proliferation as the primary measure of the study, with a minimum detectable difference of 0.1 points between the two assessment visits, a standard deviation of 1.0 (based on data from a pilot study), a statistical power of 90 %, and a significance level of 5 %, a sample size of 44 patients would be required (calculated using Minitab 16.0 software). To account for potential attrition, a total of forty-eight patients were enrolled in the current study.

### Inclusion and exclusion criteria

The inclusion criteria for the present study were as follows: meeting the ERA classification criteria outlined by the 2010 American College of Rheumatology/European League Against Rheumatism (ACR/EULAR)^17^ ; age between 18 and 65 years; female gender; and being treatment-naive. The exclusion criteria consisted of recent use of oral glucocorticoids exceeding 10 mg/day within the past three weeks; serum levels of aspartate aminotransferase or alanine aminotransferase exceeding three times the upper limit of normal; presence of bone marrow disorders; presence of autoimmune diseases other than ERA; history of lymphoproliferative or infectious diseases; and pregnancy.

### Treatment protocol

A tightly controlled therapeutic protocol, adapted in accordance with the recommendations of the Brazilian Consensus of Rheumatology,^18^ was implemented for all patients and supervised by a single-blinded rheumatologist.

The following treatment regimen was executed: patients initiated with methotrexate (MTX) at a dose of 15 mg/week, which was escalated to 25 mg/week until the 12th week. Subsequent steps were undertaken for patients exhibiting an inadequate response (DAS-28 > 3.2 and Physician's Global Assessment [PGA > 4.0]; on a scale of 0 to 10 cm). These steps included the addition of leflunomide at a dose of 20 mg/day alongside MTX at 15 mg/week from week 12 to week 24, followed by the administration of adalimumab twice a month in combination with MTX at 15 mg/week from week 24 to week 48. Additionally, the prescription of 5 mg of folic acid once a week was maintained throughout the 48-week study period. Within this duration, three instances of treatment failure were defined as follows:

• Failure 1: Failure to respond to the initial DMARD (MTX) at week 12.
• Failure 2: Failure to respond to the second DMARD (leflunomide) at week 24.
• Failure 3: Failure to respond to the first immunobiological drug (adalimumab) at week 48.

The use of diclofenac 50 mg on an as-needed basis was permitted, and an increase in the daily prednisone dose was allowed only if the increment did not exceed 5 mg per day. Joint injections were prohibited during the follow-up period. The criteria for therapeutic failure were solely based on the DAS 28 and the PGA, excluding daily doses of prednisone or diclofenac.

In this study, patients who experienced therapeutic failures during the observation period were categorized as the “case” group, while those who did not encounter therapeutic failures at the same assessment time points were assigned to the “control” group.

### Assessments

All patients underwent blinded clinical, laboratory, and ultrasound assessments at baseline, as well as after 12-, 24-, and 48-weeks. The clinical evaluation was conducted without access to laboratory tests and ultrasound findings. Likewise, the ultrasound assessment was performed in isolation from the clinical evaluation and laboratory results.

### Clinical assessment

The following clinical parameters were evaluated at each assessment time by a blinded rheumatologist:

• PGA of Disease Activity: This was measured on a scale of 0 to 10 cm.
• Brazilian Version of the Functional Subscale of the Stanford Health Assessment Questionnaire (HAQ).^19^
• Disabilities of the Arm, Shoulder, and Hand Questionnaire (DASH).^20^
• Disease Activity Score 28 (DAS28).^1^
• Simplified Disease Activity Index (SDAI).^2^
• Clinical Disease Activity Index (CDAI).^3^

### Laboratory evaluation

• Comprehensive laboratory assessments were conducted at each evaluation time, encompassing the following parameters:
• C-reactive Protein (CRP) Levels in milligrams per deciliter (mg/dL).
• Erythrocyte Sedimentation Rate (ESR): in millimeters per hour (mm/hour).
• Blood Count.
• Sérum Aspartate Aminotransferase.
• Sérum Alanine Aminotransferase.
• Gamma-Glutamyl Transferase.
• Creatinine.
• Urea.

In addition, IgM rheumatoid factor and anti-Cyclic Citrullinated Peptide (anti-CCP) antibodies were examined during the baseline assessment.

### Ultrasound assessment

The ultrasound examination was conducted by a proficient rheumatologist with a decade of experience, who remained blinded throughout the process. The assessment employed a MyLab60 ultrasound system (Esaote, Biomedica – Genoa, Italy), equipped with a broadband linear probe possessing a frequency range from 6 to 18 MHz.

A meticulous and standardized ultrasound evaluation was systematically executed, adhering to the guidelines set forth by the European League Against Rheumatism (EULAR).^21^ This evaluation occurred at all four assessment time points and encompassed an examination of ten joints (five bilaterally), as outlined below:

• Wrist: Examination of the dorsal face (greater joint recess), radiocarpal or medio carpal recess, and ulnocarpal joint recess, bilaterally.
• Second Metacarpophalangeal Joint (MCP): Evaluation of both the dorsal and volar faces.
• Third MCP Joint: Assessment of both the dorsal and volar faces.
• Second Proximal Interphalangeal (PIP) Joint: Examination of the volar face.
• Third Interphalangeal (IP) Joint: Analysis of the volar face.

The ultrasound evaluation performed on these ten joints was denoted as the US10 system. The subsequent ultrasound parameters were assessed (Table 1).

**Table 1:** US10 System Range and its values parameters of the sample at baseline.

Score range of US10 parameters at baseline											
US10	Total Score Range	Mean ± SD at T0	US MCP	MCP Score Range	Mean ± SD at T0	US PIP	PIP Score Range	Mean ± SD at T0	US wrist	Wrist Score Range	Mean±SD at T0
**Inflammation Parameters**											
SP/Q10 Total Score	**0 – 16**	12.9 ± 2.9	**SP/Q MCP**	**0 ‒ 8**	5.8 ± 1.6	**SP/Q PIP**	**0 ‒ 4**	3.2 ± 1.0	**SP/Q wrist**	**0 ‒ 4**	3.0 ± 1.9
SP/SQ10 Total Score	**0 – 48**	29.1 ± 8.4	**SP/SQ MCP**	**0 ‒ 24**	12.9 ± 4.1	**SP/SQ PIP**	**0 ‒ 12**	6.8 ± 2.6	**SP/SQ wrist**	**0 ‒ 12**	7.2 ± 2.5
PD/Q10 Total Score	**0 –16**	6.7 ± 4.1	**PD/Q MCP**	**0 ‒ 8**	3.3 ± 2.7	**PD/Q PIP**	**0 ‒ 4**	0.9 ± 1.1	**PD/Q wrist**	**0 ‒ 4**	2.0 ± 1.3
PD/SQ10 Total Score	**0 – 48**	14.2 ± 8.9	**PD/SQ MCP**	**0 ‒24**	7.0 ± 4.7	**PD/SQ PIP**	**0 ‒ 12**	2.9 ± 2.7	**PD/SQ wrist**	**0 ‒ 12**	4.3 ± 3.0
Tn/GS/Q Total Score	**0 - 10**	2.9 ± 2.5	**Tn/GS/Q MCP**	**0 ‒ 4**	0.6 ± 1.0				**Tn/GS/Q wrist**	**0 ‒ 3**	2.2 ± 1.8
Tn/PD/Q Total Score	**0 −10**	2.3 ± 2.2	**Tn/PD/Q MCP**	**0 ‒ 4**	0.6 ± 1.0				**Tn/PD/Q wrist**	**0 ‒ 3**	1.9 ± 1.8
**Joint Damage Parameters**											
BE/Q10 Total Store	**0 – 12**	4.7 ± 1.8	**BE/Q MCP**	**0 ‒ 6**	4.7 ± 0.3	**BE/Q PIP**	**0 ‒ 4**	0.20 ± 0.2	**BE/Q wrist**	**0 ‒ 2**	1.2 ± 0.9
BE/SQ10 Total Score	**0 – 36**	9.7 ± 3.8	**BE/SQ MCP**	**0 ‒ 18**	9.7 ± 0.5	**BE/SQ PIP**	**0 ‒ 12**	0.13 ± 0.5	**BE/SQ wrist**	**0 ‒ 6**	2.4 ± 1.8
CD/Q Total Score	**0 – 4**	0.2 ± 0.1									
CD/SQ Total Score	**0 – 16**	1.2 ± 1.8									

US10, Ultrasound Score of hand joints and wrist; SD, Standard Deviation; SP, Synovial Proliferation; PD, Power Doppler; Tn, Tenosynovitis; BE, Bone Erosion; CD, Cartilage Damage; SQ, Semi-Quantitative Assessment; Q, Qualitative Assessment; MCP, Metacarpophalangeal joint; PIP, Proximal Interphalangeal Joint.

### Inflammation parameters

1) Synovial Proliferation (SP): This is defined as the presence of a hypoechoic/anechoic area visible in both planes on the grey scale (GS).^22^
  a) Semi-quantitative assessment (SQ): Scored on a scale of 0–3,^23^ with a maximum score of 48.
  b) Qualitative assessment (Q): Evaluated through binary assessment: 0 (absent or Grade 1) and 1 (present, if Grade 2 or 3), with a maximum score of 16.
2) Synovial Blood Flow: Assessed using power Doppler (PD) in the same joint recesses that were evaluated for Synovial Proliferation (SP). PD settings were standardized with a pulse repetition frequency of 750 Hz and a color-mode frequency of 12 MHz. Wall filters were set to the lowest value, while color gain was increased to the highest value to prevent PD signals from being generated under the bone cortex.
  a) Semi-quantitative assessment: Scored on a scale of 0–3,^24^ with a maximum score of 48.
  b) Qualitative assessment: Evaluated through binary assessment: 0 (absent) or 1 (present, if the semi-quantitative score is Grade 1, 2, or 3), with a maximum score of 16.
3) Tenosynovitis (Tn): Assessment was performed on the following tendons ‒ extensor digitorum communis, extensor carpi ulnaris, flexor digitorum communis, second and third flexor tendons. Tenosynovitis was evaluated and graded using both the Grey Scale (GS) and Power Doppler (PD) qualitative scores.^22^
  a) Qualitative assessment with Grey Scale (GS): Evaluated through binary assessment: 0 (absent) or 1 (present), with a maximum score of 10.
  b) Qualitative assessment with PD: Evaluated through binary assessment: 0 (absent) or 1 (present), with a maximum score of 10.

### Joint damage parameters

1) Bone Erosion (BE): This is defined as the failure of the intra-articular bone cortex observed in both the transverse and longitudinal planes.^22^ The location of each erosion was documented based on the affected bone, as follows: dorsal quadrant of the second and third metacarpal head; lateral quadrant of the second metacarpal head; dorsal quadrant of the second and third PIP joints; ulnar styloid process. The severity of BEs was assessed using both a semi-quantitative and qualitative scoring system:
  a) Semi-quantitative assessment: Graded on a scale of 0‒3,^25^ with a maximum score of 36.
  b) Qualitative assessment: Evaluated through binary evaluation: 0 (absent or Grade 1) or 1 (present, if Grade 2 or 3), with a maximum score of 12.
2) Cartilage Damage (CD): Ultrasound examinations were focused on evaluating the hyaline cartilage in the dorsal view of the second and third metacarpal heads (Grassi et al. 2004). CD was assessed using the following semi-quantitative and qualitative scoring system;^26^^,^^27^
  a) Semi-quantitative assessment: Graded on a scale of 0‒4,^10^ with a maximum score of 16.
  b) Qualitative assessment: Assessed through binary evaluation: 0 (absent or Grade 1) or 1 (present, if Grade 2, 3, or 4), with a maximum score of 4.

For each of these parameters, there were total scores (maximum of 10) and scores for the three sub-items: MCP, PIP, and wrist. Exceptions were observed for parameters related to tenosynovitis, which included the total score and scores for the sub-items MCP and wrist, as well as parameters related to CD, which exclusively had the total score (Table 1).

### Statistical analysis

Statistical analysis was conducted using the SPSS program, version 17.0 (SPSS, Chicago, IL, USA). Data were presented as mean ± standard deviation. ANOVA was employed to compare repeated numerical variables across different time points. The statistical analyses of the study exclusively considered data from the baseline ultrasound assessment. The sole clinical data utilized in this analysis was the categorical indicator (yes or no) of whether the patient experienced therapeutic failure.

The ROC curve was generated to determine the threshold values for baseline ultrasound variables that could predict therapeutic failures 1, 2, and 3, along with corresponding values for sensitivity, specificity, positive and negative predictive values, and accuracy.

Following the identification of cut-off values through the ROC curve, Multivariate Logistic Regression analysis was conducted. This analysis assessed the Odds Ratio associated with each cut-off value in the baseline ultrasound measurements for predicting therapeutic failures. The analysis was conducted separately for therapeutic failures 1, 2, and 3. Only the values obtained from the baseline ultrasound assessment were included in the multivariate logistic regression analysis, without the inclusion of any other clinical or laboratory parameters. The level of statistical significance was set at 5 % (*p* < 0.05).

An analysis of interobserver reproducibility was conducted by an experienced sonographer who evaluated 10 % of our patient sample independently. After the first sonographer completed the ultrasound evaluation, he left the room, and the second sonographer entered and conducted his evaluation using a separate assessment sheet. Our interobserver reproducibility was performed using the Kappa values following this categorization: excellent (> 0.81), substantial (0.61‒0.80), moderate (0.41‒0.60), good (0.21‒0.40), minimum (0.20‒0), and not agreeing (≤ 0).^27^

## Results

Forty-eight women with a mean age of 47.7 ± 11.6-years and a mean disease duration of 7.5 ± 3.5-months were included. Rheumatoid factor and anti-CCP were positive in 41.7 % and 43.8 % of the participants, respectively (39.58 % were double positives), with 43.75 % of them using oral corticosteroids.

All patients strictly adhered to the treatment protocol recommended at the beginning of the study. There was no utilization of other types of treatment or interventions that deviated from this protocol. The baseline data for the sample are detailed in Table 2. Of the participants, 41 (85.41 %) experienced therapeutic failure 1, 25 patients (52 %) experienced therapeutic failure 2, and only 5 patients (10.5 %) experienced therapeutic failure 3. All patients who experienced any of the three therapeutic failures had a DAS-28 score greater than 3.2 and an AGM greater than 4.

**Table 2:** Demographic and clinical characteristics of RA patients at baseline.

**Number of patients**	48
**Age in years**	47.7 ± 11.6
**Gender F/M**	48 (100 %)
**Disease time ‒ months**	7.5 ± 3.5
**Rheumatoid Factor+**	20 (41.7 %)
**Anti – CCP+**	21 (43.8 %)
**Rheumatoid Factor+ / Anti-CCP+**	19 (39.58 %)
**Oral corticosteroid**	21 (43.75 %)
**Prednisone (mg/dia)**	3.2 ± 4.2
**DAS 28**	6.5 ± 1.3
**SDAI**	46.4 ± 16.5
**CDAI**	44.9 ± 15.9
**HAQ**	1.34 ± 0.67
**DASH**	48.14 ± 23.54
**ESR (mm/h)**	29.7 ± 24.2
**CRP (mg/dL)**	13.5 ± 17.6

Data presented as mean ± standard deviation or n (%); F, Female; M, Male; DAS28, 28-Joint Disease Activity Score; SDAI, Simplified Disease Activity Score; CDAI, Clinical Disease Activity Index CDAI; HAQ, Stanford Health Assessment Questionnaire; DASH, Disabilities of the Arm. Shoulder and Hand Questionnaire; ESR, Erythrocyte Sedimentation Rate; CRP, C-Reactive Protein.

The mean total scores for SP, PD, Tn/GS, Tn/PD, BE, and CD at T0 were 12.9 (±2.9), 6.7 (±4.1), 2.9 (±2.5), 2.3 (±2.2), 4.7 (±1.8), and 0.2 (±0.1) respectively (as shown in Table 1). Table 1 indicates that at T0, the lowest scores proportionally were recorded for joint CD, while the highest scores were recorded for SP variables. Additionally, it is observed that at T0, some of these patients already exhibited some degree of BE, mainly in the MCPs.

### US10 parameters and their sub-items capable of predicting therapeutic failures

Table 1 displays the US10 parameters at baseline. The patients underwent ultrasonographic evaluation, which demonstrated statistical improvement (*p* < 0.01) in the scores of SP and tenosynovitis, primarily from T0 to T12 (Table 3). Persistent decreases (*p* < 0.01) were noted in PD scores from T0 to T48. However, BE and CD scores (qualitative measurements) exhibited an increase during the study (*p* = 0.01). Meanwhile, parameters related to clinical and functional assessments consistently decreased (*p* < 0.01) from T0 to T48, except for ESR and CRP, for which the decline over time was not statistically significant (Table 3).

**Table 3:** US10, clinical and laboratory parameters during the 48-weeks of the study.

US10 total score parameters															
Time-points weeks	Inflammation parameters									Joint damage parameters					
	SP/Q10	SP/SQ10		PD/Q10		PD/SQ10		Tn/GS/Q	Tn/PD/Q	BE/Q10	BE/SQ10		CD/Q		CD/SQ
T0	12.9 ± 1.3	29.1 ± 8.4		6.7 ± 4.1		14.2 ± 3.8		2.9 ± 2.5	2.3 ± 2.2	4.7 ± 1.8	9.7 ± 0.8		0.2 ± 0.5		1.2 ± 1.8
T12	4.2 ± 1.3	10.3 ± 8.7		2.7 ± 2.5		5.0 ± 5.1		1.2 ± 2.0	1.1 ± 1.7	5.1 ± 2.0	10.9 ± 4.4		0.6 ± 1.3		1.7 ± 3.1
T24	8.4 ± 1.3	17.3 ± 6.8		2.3 ± 2.1		44.2 ± 3.9		1.7 ± 2.0	1.4 ± 1.9	5.9 ± 1.4	12.1 ± 2.9		1.0 ± 1.3		2.4 ± 3.3
T48	7.0 ± 1.3	14.2 ± 7.9		0.7 ± 1.3		1.2 ± 2.3		0.6 ± 2.4	0.3 ± 1.0	6.0 ± 1.9	12.7 ± 3.8		1.1 ± 1.3		2.5 ± 3.6
p	**<0.01**	**<0.01**		**<0.01**		**<0.01**		**<0.01**	**<0.01**	**<0.01**	**0.01**		**<0.01**		**0.146**
**Clinical and laboratory parameters**															
	**DAS28**		**SDAI**		**CDAI**		**PGA**		**HAQ**	**DASH**		**CRP**		**ESR**	
T0	6.5 ± 1.3		46.8 ± 16.2		45.2 ± 15.5		6.1 ± 2.1		1.38±0.68	47.98±23.2		14.0 ± 18.0		30.6 ± 3.5	
T12	4.5 ± 1.7		26.3 ± 16.3		23.2 ± 1.7		3.7 ± 2.2		0.7 ± 0.55	23.78±20.1		8.4 ± 11.4		21.9 ± 2.8	
T24	4.7 ± 1.6		24.1 ± 17.4		23.4 ± 17.3		3.6 ± 2.3		0.85±0.68	27.18±21.8		7.8 ± 8.9		24.0 ± 2.6	
T48	3.9 ± 1.4		15.0 ± 13.1		15.3 ± 15.2		2.7 ± 1.9		0.7 ± 0.6	25.06±24.2		6.9 ± 12.1		24.3 ± 2.3	
p	<0.01		<0.01		<0.01		<0.01		<0.01	<0.01		0.325		0.058	

US10, Ultrasound score of hand joints and wrists; Data presented as mean ± standard deviation; SP, Synovial Proliferation; PD, Power Doppler; Tn, Tenosynovitis; BE, Bone Erosion; CD, Cartilage Damage; SQ, Semi-Quantitative assessment; Q, Qualitative assessment; MCP, Metacarpophalangeal joint; PIP, Proximal Interphalangeal joint; DAS28, 28-Joint Disease Activity Score; SDAI, Simplified Disease Activity Score; CDAI, Clinical Disease Activity Index; PGA, Physician-based Global Assessment of disease activity 0‒10 cm; HAQ, Stanford Health Assessment Questionnaire; DASH, Disabilities of the Arm, Shoulder and Hand Questionnaire; ESR, Erythrocyte Sedimentation Rate; CRP, C-reactive protein; Statistical Test, ANOVA for repeated measurements.

Several items and sub-items of the US10 system at T0 were identified as predictors of therapeutic failures, particularly Failure 1 and Failure 2. These results were observed in both the ROC Curve analysis and the Multivariate Logistic Regression analysis.

### Analysis of the ROC curve

**Fig. 1:**
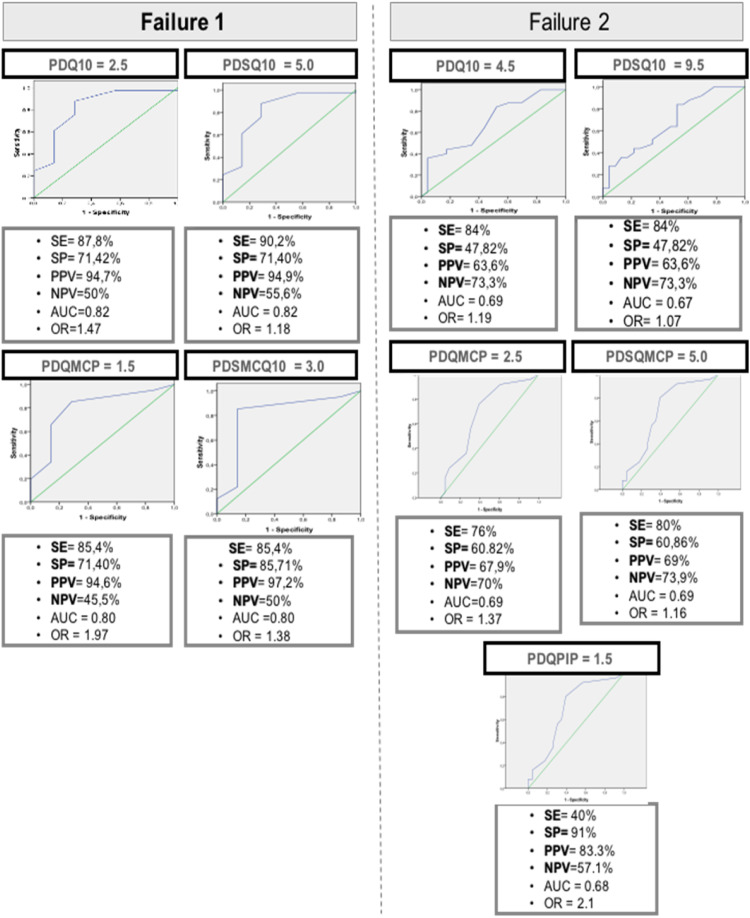
US-10 values at T0, predictors of failure in the first DMARD (Failure 1) and in the second DMARD (Failure 2), according to the ROC Curve. AUC, Under Area Curve; SE, Sensitivity; SP, Specificity; PPV, Positive Predictive Value; NPV, Negative Predictive Value; PD, Power Doppler; SQ, Semi-Quantitative assessment; Q, Qualitative assessment; MCP, Metacarpophalangeal joint; PIP, Proximal Interphalangeal Joint; DMARD, Disease Modifying Antirheumatic Drug.

Table 4 and Fig. 1 depict the ultrasound variables at T0 that predict Failures 1, 2, and 3, as determined by the ROC curve analysis, along with their corresponding sub-items. Regarding Failure 1, the following predictors were identified: PD/Q10 total score > 2.5; PD/SQ10 total score > 5; PD/Q MCP > 1.5; and PD/SQ MCP > 3. Notably, the PD/SQ MCP > 3 variable emerged as the most robust predictor for Failure 1. For Failure 2, similar predictors were observed, albeit with higher sonographic scores, along with the addition of the sub-item PD/Q PIP. These predictors included: PD/Q10 total score > 4.5; PD/SQ10 total score > 9.5; PD/Q MCP > 2.5; PD/SQ MCP > 5; and PD/Q PIP > 1.5. In this case, the PD/Q PIP > 1.5 variable stood out as the most effective predictor for Failure 2. Failure 3 was predicted by only one ultrasound variable: PD/Q wrist > 2.5.

**Table 4:** US10 items and subitems in T0 to predict therapeutic failure according to the analysis of the ROC curve.

Analysis of the ROC Curve								
US-10 parameters	AUC	p	Cutoff Value	SNS (%)	SP (%)	NPV (%)	PPV (%)	Accuracy %
**Failure 1 – failure to the first DMARD**								
PD/Q10 total score	0.82	0.012	>2.5	87.80	71.42	50.00	94.70	85.40
PD/SQ10 total score	0.81	0.010	>5.0	90.20	71.40	55.60	94.90	87.50
PD/Q MCP	0.80	0.033	>1.5	85.40	71.40	45.50	94.60	83.30
PD/SQ MCP	0.80	0.007	>3.0	85.40	85.71	50.00	97.20	85.40
**Failure 2 – failure to the first and second DMARDs**								
PD/Q10 total scsore	0.69	0.023	>4.5	84.00	47.82	73.30	63.60	66.70
PD/SQ10 total score	0.67	0.035	>9.5	84.00	47.82	73.30	63.60	66.70
PD/Q MCP	0.69	0.022	>2.5	76.00	60.82	70.00	67.90	68.70
PD/SQ MCP	0.69	0.021	>5.0	80.00	60.86	73.70	69.00	70.83
PD/Q PIP	0.67	0.048	>1.5	40.00	91.00	57.10	83.30	62.50
**Failure 3 – failure to the first and second DMARDs and to the first immunobiological drug**								
PD/Q wrist	0.79	0.035	>2.5	100.00	65.00	100.0	25.00	68.00

AUC, Under Area Curve; SNS, Sensitivity; SP, Specificity; NPV, Negative Predictive Value; PPV, Positive Predictive Value; PD, Power Doppler; SQ, Semi-Quantitative assessment; Q, Qualitative assessment; MCP, Metacarpophalangeal joint; PIP, Proximal Interphalangeal Joint; DMARD, Disease Modifying Antirheumatic Drug.

As evidenced in Table 4 and Fig. 1, among the ultrasound variables predicting failure, PD/SQ MCP (Failure 1) exhibited the most pronounced statistical significance. The variable with the highest Area Under the Curve (AUC) was the PD/Q10 total score (Failure 1). PD/Q wrist emerged as the variable with the greatest sensitivity (Failure 3), while PD/Q PIP demonstrated the highest specificity (Failure 2). The PD/Q wrist recorded the most notable negative predictive value (Failure 3), whereas the PD/SQ MCP displayed the highest positive predictive value (Failure 1). Notably, the ultrasound variable that achieved the highest accuracy was the PD/SQ10 total score (Failure 1). It is worth highlighting the variable PD/Q wrist, which exclusively predicted Failure 3 with 100 % sensitivity and 100 % negative predictive value. Due to its sensitivity of 100 %, the curve for PD/Q wrist could not be represented in Fig. 1.

### Multivariate logistic regression analysis

Table 5 presents the cutoff values at T0 for variables predicting failures along with their respective Odds Ratios (OR). For Failure 1, the predictive variables were as follows: PD/Q10 total score > 2.5 (OR = 18); PD/SQ10 total score > 5 (OR = 23.12); PD/Q MCP > 1.5 (OR = 14.58); and PD/SQ MCP > 3 (OR = 35). The variable PD/SQ MCP > 3 emerged as the most reliable predictor for Failure 1. Concerning Failure 2, the predictors were: PD/Q10 total score > 4.5 (OR = 4.81); PD/SQ10 total score > 9.5 (OR = 4.81); PD/Q MCP > 2.5 (OR = 4.92); PD/SQ MCP > 5 (OR = 6.22); and PD/Q PIP > 1.5 (OR = 6.66). In the context of Failure 2, the variable PD/Q PIP > 1.5 demonstrated the strongest predictive capability (Table 5). A sole ultrasound variable, PD/Q wrist > 2.5, predicted Failure 3; nevertheless, it was not feasible to compute the OR for this variable due to the cutoff value yielding a sensitivity of 100 %.

**Table 5:** Prediction of US10 items and sub-items in T0 for therapeutic failures according to the Multivariate Logistic Regression analysis.

Multivariate logistic regression analysis			
US 10 - Parameters	OR	95 % CI	p
**Failure 1 – failure to the first DMARD**			
PD/Q10 total score > 2.5	18.00	2.72 ‒ 118.94	0.003
PD/SQ10 total score > 5.0	23.12	3.32 ‒ 160.49	0.001
PD/Q MCP score > 1.5	14.58	2.28 ‒ 93.16	0.005
PD/SQ MCP score > 3.0	35.00	3.55 ‒ 344.68	0.002
**Failure 2 – failure to the first and second DMARDs**			
PD/Q10 total score > 4.5	4.81	1.25 ‒ 1.84	0.022
PD/SQ10 total score > 9.5	4.81	1.25 ‒ 1.84	0.022
PD/Q MCP score > 2.5	4.92	1.42 ‒ 17.06	0.012
PD/SQ MCP score > 5.0	6.22	1.71 ‒ 22.58	0.005
PD/Q PIP score > 1.5	6.66	1.26 ‒ 35.03	0.025
**Prediction for each unit added to the items and sub-items of the US10 in T0 for therapeutic failures 1 and 2**			
**Failure 1 – failure to the first DMARD**			
PD/Q10 total score	1.47	1.04 ‒ 1.47	0.026
PD/SQ10 total score	1.18	1.01 ‒ 1.38	0.030
PD/Q MCP score	1.97	1.05 ‒ 3.69	0.033
PD/SQ MCP score	1.38	1.02 ‒ 1.86	0.033
**Failure 2 – failure to the first and second DMARDs**			
PD/Q10 total score	1.19	1.01 ‒ 1.19	0.028
PD/SQ10 total score	1.07	1.00 ‒ 1.15	0.044
PD/Q MCP score	1.37	1.02 ‒ 1.85	0.037
PD/SQ MCP score	1.16	1.01 ‒ 1.38	0.034
PD/Q PIP score	2.1	1.09 ‒ 4.38	0.027

OR, Odds Ratio; IC, Confidence Interval; SP, Synovial Proliferation; PD, Power Doppler; Tn, Tenosynovitis; BE, Bone Erosion; CD, Cartilage Damage; SQ, Semi-Quantitative assessment; Q, Qualitative assessment; MCP, Metacarpophalangeal joint; PIP, Proximal Interphalangeal Joint; DMARD, Disease Modifying Antirheumatic Drug.

In the multivariate logistic regression analysis, the Odds Ratio was also identified for predicting therapeutic failure per unit of ultrasound score at T0 for items and sub-items of the US10 that had been earlier established as predictors of therapeutic failure (Table 5). Through this calculation, it was noted that the optimal predictor for Failure 1 shifted to the item PD/Q MCP (OR = 1.97). The item PD/Q PIP sustained its role as the prime predictor of Failure 2 (OR = 2.1). While a trend towards predicting Failure 3 was observed for each additional unit of ultrasound measurement of PD/Q wrist (OR = 2.72), statistical significance was not achieved (*p* = 0.058).

### Analysis of inter-observer reproducibility

The assessment of interobserver reproducibility within the study, conducted on 10 % of the sample using the Kappa test (κ), yielded the following outcomes: SP/Q total score: κ = 0.499 (*p* < 0.000); SP/SQ total score: κ = 0.215 (*p* = 0.014); PD/Q total score: κ = 0.492 (*p* < 0.000); PD/SQ total score: κ = 0.569 (*p* < 0.000); Tn/GS/Q total score: κ = 0.551 (*p* < 0.000); Tn/PD/Q total score: κ = 0.324 (*p* < 0.000); BE/Q total score: κ = 0.429 (*p* < 0.000); BE/SQ total score: κ = 0.471 (*p* < 0.000); CD/Q: κ = 0.793 (*p* < 0.000) and CD/SQ: κ = 0.828 (*p* < 0.000).

## Discussion

This study aimed to identify ultrasound parameters within the US10 scoring system capable of predicting therapeutic failures in treatment-naive women with Early Rheumatoid Arthritis (ERA) followed over 48 weeks.

Our findings revealed that Power Doppler (PD) measurements could predict therapeutic failures not only for the first and second Disease-Modifying Antirheumatic Drugs (DMARDs), but also for the initial immunobiological drug. Both components of the US10 total score (PD/Q10, PD/SQ10), along with specific sub-items (PD/Q MCP, PD/SQ MCP, PD/Q PIP, and PD/Q wrist), emerged as predictive of therapeutic failure.

The value of ultrasound in monitoring RA patients and influencing treatment changes is established.^28^ When the authors began our study, there were no published studies assessing whether baseline ultrasound changes could predict “therapeutic failure” in patients with ERA after one year. Even today, the authors have not found a study with the exact same design.

Our results align with Valor et al.'s findings (2018) which identified predictors of immunobiological drug failure at 40 months, including DAS28 > 2.2, PD, rheumatoid factor, and smoking in 77 RA patients assessed via ultrasound in 42 joints.^14^ This underscores PD's significance as a predictor of poor outcomes in these patients.

Conversely, our results contrast with two larger-scale studies.^15^^,^^16^ Bergstra et al.'s study (2019) involving 4623 patients found no association between combined Anti-Citrullinated Protein Antibodies (ACPA) presence and bone erosions on ultrasound with treatment response after 6 to 12 months.^15^ Similarly, Ten Cate et al. (2018), studying 159 patients, found PD and bone erosions in hand and foot ultrasound not predictive of achieving remission in 12 months; instead, high DAS28 and rheumatoid factor were predictors.^16^ These studies' larger sample sizes and broader ultrasound coverage could contribute to the differing outcomes.

The present study noted statistical improvement in all inflammatory US10 parameters over 48 weeks. Similar observations were made in other ERA studies without DMARD usage. PD changes in 28 joints showed a 12-month follow-up improvement in active joint counts and total PD scores.^29^ Another study indicated that grey-scale synovitis and PD remained detectable in 95 % and 41 % of patients, respectively, after 12-months.^30^ Backhaus et al. reported statistical improvement in synovial and tenosynovitis proliferation after 3 and 6 months using the US7 score in various arthritis types.^31^

Interestingly, this study revealed distinct wrist and small hand joint ultrasound evolution patterns during follow-up. This aligns with Ribbens et al.'s findings (2003) which showed a higher SP measure improvement in MCPs, and PIPs (80 %) compared to wrists (60 %) after 6-weeks of anti-TNFα treatment in RA patients.^32^ This disparity likely results from more substantial pannus volume in the wrist than in small hand joints.

Unlike SP, our study demonstrated a consistent PD/Q10 and PD/SQ10 total score decrease across all assessments after baseline. Studies also indicate PD reduction in the hands following various RA treatments, including adalimumab and infliximab, sometimes even within the first weeks.^33^, ^34^^,^^35^ Notably, our data highlighted PD improvement as early as the 12th week after MTXintroduction. This echoes Hammer and Kvien's study (2011) which found synovial and tenosynovitis proliferation improvement at the 12-month mark post-adalimumab introduction.^36^

In the realm of RA treatment, numerous patients fail DMARDs and initial immunobiological drugs. The present study aimed to establish an association between baseline hand ultrasound measurements and these therapeutic failures. Our surprising results deserve elaboration. The authors revealed that PD/Q10 total score, PD/SQ10 total score, PD/Q MCP, and PD/SQ MCP could predict Failure 1. These same ultrasound parameters, featuring higher scores alongside PD/Q PIP even at low levels (> 1.5), predicted both Failure 1 and Failure 2. In essence, higher PD scores within the US10 total score and sub-items in MCPs, and even lower PD scores in PIPs, emerged as predictors of requiring an immunobiological drug among our patients.

Our ROC curve identified PD/Q wrist as a predictor of Failure 3. However, this was not confirmed by multivariate logistic regression analysis due to the inability to calculate its odds ratio with 100 % sensitivity. A variable with 100 % sensitivity can lead to perfect collinearity, which compromises the model's ability to accurately estimate the effects of the independent variables and interpret the results. This unusual data suggests that even intermediate PD wrist scores (PD/Q wrist > 2.5) in ERA patients could signal a high likelihood of necessitating a second immunobiological drug. /

Notably, the literature lacks joint ultrasound variables as predictive of negative outcomes as PD. PD has long demonstrated the ability to predict future joint damage, even in cases of subclinical synovitis, disease relapse during clinical remission, and failure to discontinue immunobiological therapy during remission.^14^^,^^15^^,^^37^, ^38^, ^39^ A systematic review by Ten Cate et al. noted PD's predictive potential for joint damage and disease relapse across MCPs, wrists, and MTPs.^15^ Nguyen et al.'s meta-analysis (2014) further supported PD's predictive power for disease relapse (OR = 3.2), joint damage per patient (OR = 6.9), and per joint (OR = 9.1).^39^ However, few studies have examined PD's relationship with future therapeutic failures in ERA treatment.

In line with existing literature, our results reaffirm PD as the paramount joint ultrasound variable for predicting adverse outcomes. It can identify ERA patients likely to respond poorly to recommended treatments even before their initiation. This discovery may reshape how rheumatologists approach ERA patients, potentially leading to more aggressive treatment for those with high PD scores in their hands.

The present study's inclusion of 48 ERA patients without prior MTX use was a challenging feat. Consequently, fibromyalgia was not an exclusion criterion. Given fibromyalgia's potential to skew DAS28 calculations,^40^ the authors defined “therapeutic failure” as DAS28 > 3.2 concurrent with PGA > 4.

Enrolling such patients in a single center led to a unique population with low response rates to first-line drugs (14.59 % to MTX, 48 % to leflunomide) and second-line immunobiological drug indications within 48 weeks.

What caught our attention in this study was the high rate of type 1 failure, specifically with MTX monotherapy. Type 2 failure was less frequent, and type 3 failure was even less common. This suggests that, in our sample, the combination of two synthetic DMARDs was superior to using just one, and the addition of an immunobiological agent provided even greater benefits than the initial two treatments.

These results differ from the findings of Bergstra et al. (2017),^41^ which indicated that there were no significant long-term benefits when comparing individuals with RA who started treatment with MTX monotherapy to those receiving combined therapy with prednisone or infliximab. The discrepancy between our results and theirs may be attributed to several factors in our sample, such as its smaller size, the fact that all participants were female, and, notably, the high DAS-28 score (6.5 ± 1.3) at the start of the study. In other words, the characteristics of our sample may have contributed to a poorer response to MTX monotherapy.

The distinctiveness of our sample, marked by low first two synthetic drug responses and second biologic drug prescriptions within a year, may partly account for the sustained moderate disease activity at T48.

This study has some limitations. A larger patient cohort could enhance its statistical power. However, recruiting ERA patients with less than a year of disease progression and no prior treatment from a single center proved challenging. Despite random patient recruitment, only women participated during the enrollment period. This female-only sample limits our conclusions to that gender. The authors deemed it important to include the physician's global assessment > 4 as a mandatory criterion for therapeutic failure. However, this is not a validated tool. The authors also omitted foot joints from our study to maintain practicality and to reduce the potential for mechanical overload affecting the ultrasound readings. Additionally, the authors excluded baseline clinical parameters from our statistical analysis, representing another limitation.

The present results ultimately emphasize PD's role as a premier ultrasound predictor of severe outcomes. It can anticipate therapeutic failure in patients with ERA, even prior to treatment initiation. This discovery may reshape the way rheumatologists approach ERA treatment, encouraging more aggressive management for individuals exhibiting high PD scores in their hands.

## Conclusion

In summary, the PD scores within the US10 system in this study effectively predicted therapeutic failure during the initial and subsequent stages of treatment, encompassing the first and second DMARDs, and extending to the first use of immunobiological drugs in treatment-naive women with Early Rheumatoid Arthritis (ERA) over a 48-week period. Further investigations with a larger patient cohort conducted under similar circumstances are essential to validate our findings.

## Ethics approval and consent to participate

The study received approval from the institutional review board (CEP 1061/08), and all participants provided written informed consent.

## Consent for publication

Not applicable.

## Authors’ contributions

Luz KR, Natour J, Pinheiro MM, Furtado RNV contributed to the study's conception, design, data analysis, and interpretation. Luz KR, Pinheiro MM, Petterle GS, Santos MF, Fernandes ARC participated in data acquisition. Luz KR drafted the article. All authors (Luz KR, Natour J, Pinheiro MM, Petterle GS, Santos MF, Fernandes ARC, Furtado RNV) revised the article critically for significant intellectual content and granted final approval of the version to be submitted.

## Funding

This research did not receive any specific grant from funding agencies in the public, commercial, or not-for-profit sectors.

## Declaration of competing interest

The authors declare no conflicts of interest.
